# Supplementary material for: The Role of Food Security in Mediterranean Diet Adherence Among Adolescents: Findings from the EHDLA Study
Source: Foods. 2025 Jan 27;14(3):414. doi: 10.3390/foods14030414 (PMC11816826; doi:10.3390/foods14030414)
Supplement: Supplementary file 1 [file foods-14-00414-s001.zip › foods-3393171-supplementary.pdf]

foods

MDPI

Article

The Role of Food Security in Mediterranean Diet Adherence Among Adolescents: Findings from the EHDLA Study

Andrea Aquino-Blanco [1], Estela Jiménez-López [1,2], Desirée Victoria-Montesinos [3], Héctor Gutiérrez-Espinoza [4], Jorge Olivares-Arancibia [5], Rodrigo Yañéz-Sepúlveda [6], Nerea Martín-Calvo [7,8,9] and José Francisco López-Gil [10,*]

[1] Health and Social Research Center, Universidad de Castilla-La Mancha, 16071 Cuenca, Spain
[2] Centro de Investigación Biomédica en Red de salud Mental, Instituto de Salud Carlos III, 28029 Madrid, Spain
[3] Faculty of Pharmacy and Nutrition, UCAM Universidad Católica San Antonio de Murcia, 30107 Murcia, Spain
[4] Faculty of Education, Universidad Autónoma de Chile, Santiago 7500912, Chile
[5] AFySE Group, Research in Physical Activity and School Health, School of Physical Education, Faculty of Education, Universidad de Las Américas, Santiago 7500975, Chile
[6] Faculty Education and Social Sciences, Universidad Andres Bello, Viña del Mar 2520000, Chile
[7] Department of Preventive Medicine and Public Health, Facultad de Medicina, Universidad de Navarra, 31008 Pamplona, Spain
[8] Instituto de Investigación Sanitaria de Navarra (IdiSNA), 31008 Pamplona, Spain
[9] Pathophysiology of Obesity and Nutrition, Centro de Investigación Biomédica en Red, Instituto de Salud Carlos III, 28029 Madrid, Spain
[10] One Health Research Group, Universidad de Las Américas, Quito 170124, Ecuador
* Correspondence: josefranciscolopezgil@gmail.com

Supplementary material

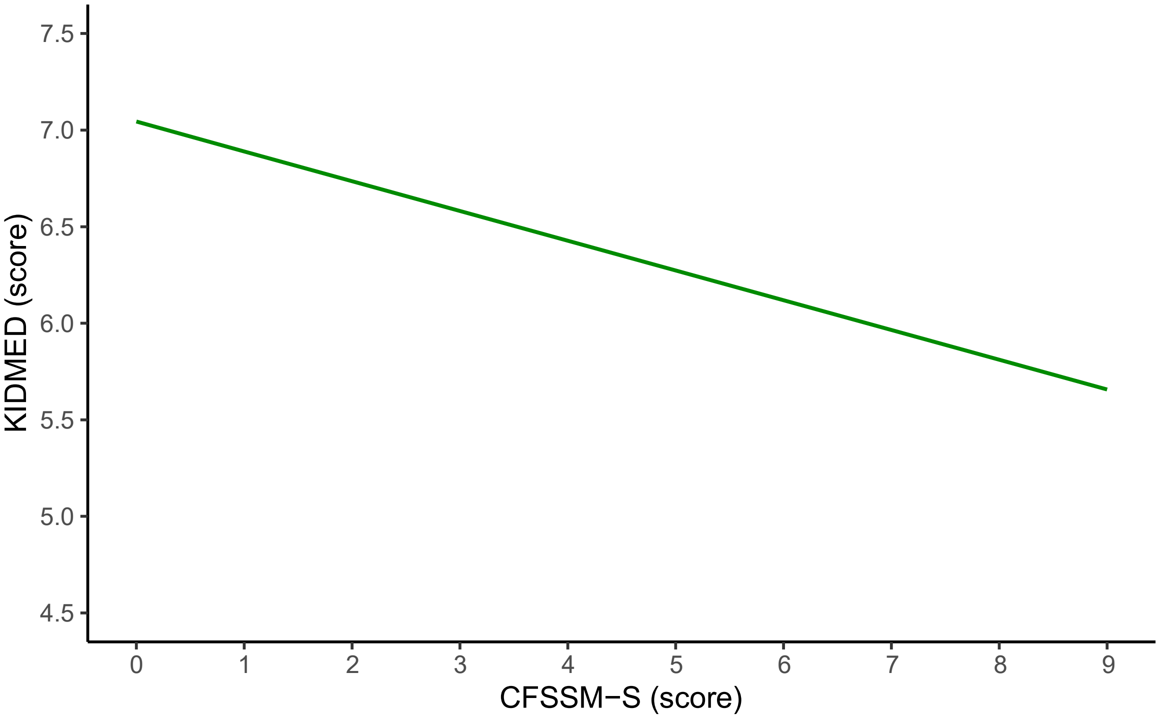

Figure S1. Estimated marginal means of the Mediterranean Diet Quality Index in children and adolescents score based on the Spanish Child Food Security Survey Module score among Spanish

adolescents. Socioeconomic status, energy intake, age, sex, physical activity, sleep duration, sedentary behavior, and body mass index were adjusted for. CFSSM-S, Spanish Child Food Security Survey Module; KIDMED, Mediterranean Diet Quality Index in children and adolescents.

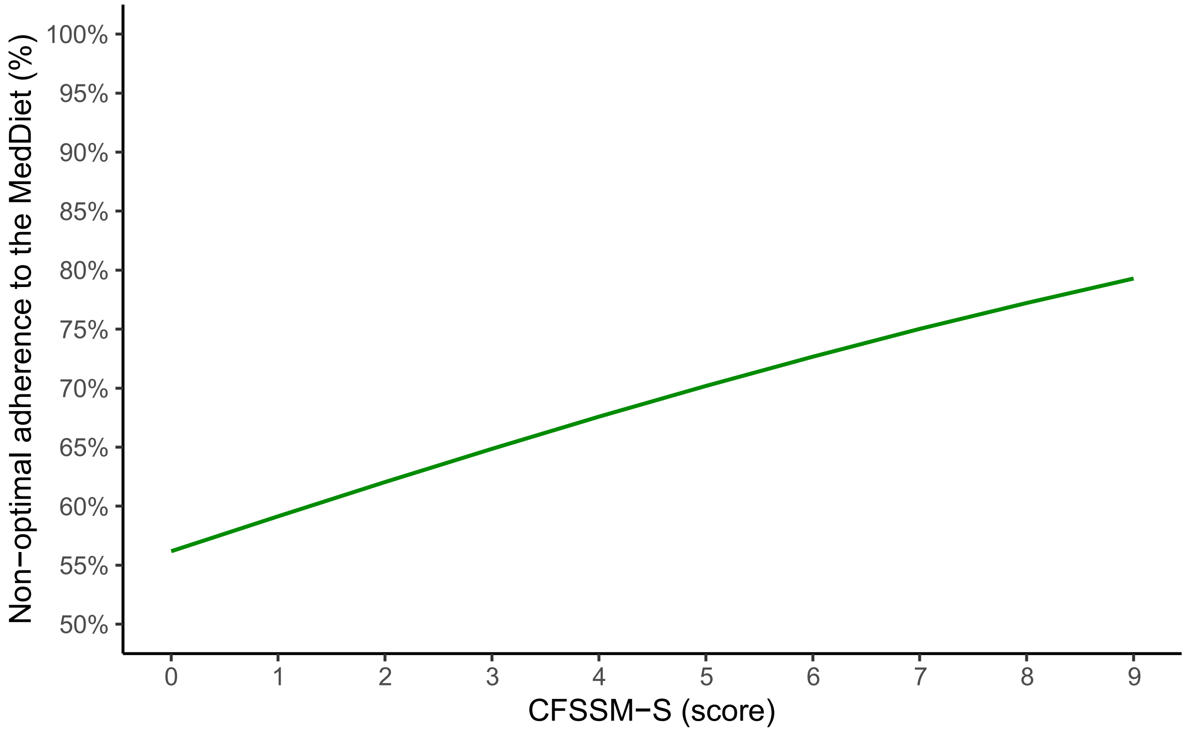

Figure S2. Predictive probabilities of nonadherence to the Mediterranean diet on the basis of the Spanish Child Food Security Survey Module score among Spanish adolescents. Socioeconomic status, energy intake, age, sex, physical activity, sleep duration, sedentary behavior, and body mass index were adjusted for. CFSSM-S, Spanish Child Food Security Survey Module; MedDiet, Mediterranean diet.

Table S1. Association of the Spanish Child Food Security Survey Module score and covariates with the Mediterranean Diet Quality Index in children and adolescents score in Spanish adolescents.

Predictor	*B*	SE	LLCI	ULCI	*p* value
CFSSM−S (per point)	-0.15	0.06	-0.26	-0.04	0.006
Age (per year)	0.15	0.06	0.04	0.26	0.009
Boys	Reference				
Girls	-0.53	0.17	-0.86	-0.19	0.002
FAS-III score (per point)	0.05	0.04	-0.03	0.13	0.201
Body mass index (per kg/m^2)	0.01	0.02	-0.02	0.05	0.444
Overall sleep duration (per hour)	0.27	0.10	0.08	0.47	0.006
YAP-S physical activity (per point)	0.69	0.13	0.44	0.94	<0.001
YAP-S sedentary behaviors (per point)	-0.80	0.14	-1.08	-0.52	<0.001
Energy intake (per 1000 kcal)	0.14	0.04	0.06	0.23	0.001

B, unstandardized beta coefficient; FAS, Family Affluence Scale-III; YAP-S, Spanish Youth Active Profile; SE, standard error; LLCI, lower limit confidence interval; ULCI, upper limit confidence interval. Socioeconomic status, energy intake, age, sex, physical activity, sleep duration, sedentary behavior, and body mass index were adjusted for.

Table S2. Association of food security status and covariates with the Mediterranean Diet Quality Index in children and adolescents in Spanish adolescents.

Predictor	*B*	SE	LLCI	ULCI	*p* value
Food secure (0-1 point)	Reference				
Low food security (2-5 points)	-0.23	0.25	-0.72	0.26	0.351
Very low food security (6-9 points)	-1.05	0.51	-2.06	-0.05	0.040
Age (per year)	0.15	0.06	0.04	0.26	0.010
Boys	Reference				
Girls	-0.52	0.17	-0.86	-0.18	0.003
FAS-III score (per point)	0.06	0.04	-0.02	0.14	0.132
Body mass index (per kg/m^2)	0.01	0.02	-0.02	0.05	0.481
Overall sleep duration (per minute)	0.28	0.10	0.08	0.48	0.005
YAP-S physical activity (per point)	0.68	0.13	0.43	0.93	<0.001
YAP-S sedentary behaviors (per point)	-0.80	0.14	-1.08	-0.52	<0.001
Energy intake (per 1000 kcal)	0.14	0.04	0.05	0.22	0.001

B, unstandardized beta coefficient; FAS, Family Affluence Scale-III; YAP-S, Spanish Youth Active Profile; SE, standard error; LLCI, lower limit confidence interval; ULCI, upper limit confidence interval. Socioeconomic status, energy intake, age, sex, physical activity, sleep duration, sedentary behavior, and body mass index were adjusted for.

Table S3. Association of the Spanish Child Food Security Survey Module score and covariates with nonoptimal adherence to the Mediterranean diet in Spanish adolescents.

Predictor	OR	SE	LLCI	ULCI	*p* value
CFSSM−S (per point)	1.13	0.06	1.01	1.26	0.031
Age (per year)	0.89	0.05	0.80	0.98	0.023
Boys	Reference				
Girls	1.43	0.16	1.05	1.95	0.023
FAS-III score (per point)	1.02	0.04	0.95	1.09	0.581
Body mass index (per kg/m^2)	0.99	0.02	0.96	1.02	0.564
Overall sleep duration (per hour)	0.80	0.10	0.66	0.97	0.020
YAP-S physical activity (per point)	0.59	0.12	0.47	0.75	<0.001
YAP-S sedentary behaviors (per point)	1.93	0.14	1.47	2.54	<0.001
Energy intake (per 1000 kcal)	0.86	0.05	0.78	0.94	0.001

OR, odds ratio; CFSSM-S, Spanish Child Food Security Survey Module; LLCI, lower limit confidence interval; SE, standard error; ULCI, upper limit confidence interval; FAS-III, Family Affluence Scale-III; YAP-S, Spanish Youth Active Profile. Socioeconomic status, energy intake, age, sex, physical activity, sleep duration, sedentary behavior, and body mass index were adjusted for.

Table S4. Association of food security status and covariates with nonoptimal adherence to the Mediterranean diet in Spanish adolescents.

Predictor	OR	SE	LLCI	ULCI	*p* value
Very low food security (6-9 points)	Reference				
Low food security (2-5 points)	1.20	0.23	0.76	1.90	0.438
Food secure (0-1 point)	2.55	0.54	0.89	7.36	0.083
Age (per year)	0.89	0.05	0.80	0.98	0.024
Boys	Reference				
Girls	1.42	0.16	1.04	1.94	0.026
FAS-III score (per point)	1.01	0.04	0.94	1.09	0.720
Body mass index (per kg/m^2)	0.99	0.02	0.96	1.02	0.576
Overall sleep duration (per hour)	0.80	0.10	0.66	0.96	0.019
YAP-S physical activity (per point)	0.60	0.12	0.47	0.76	<0.001
YAP-S sedentary behaviors (per point)	1.94	0.14	1.48	2.55	<0.001
Energy intake (per 1000 kcal)	0.86	0.05	0.78	0.94	0.001

OR, odds ratio; CFSSM-S, Spanish Child Food Security Survey Module; LLCI, lower limit confidence interval; SE, standard error; ULCI, upper limit confidence interval; FAS-III, Family Affluence Scale-III; YAP-S, Spanish Youth Active Profile. Socioeconomic status, energy intake, age, sex, physical activity, sleep duration, sedentary behavior, and body mass index were adjusted for.
